# Mediating Role of Self-Efficacy in the Relationship Between Optimism, Psychological Well-Being, and Resilience Among Iranian Students

**DOI:** 10.3389/fpsyg.2021.675645

**Published:** 2021-06-14

**Authors:** Fatemeh Sabouripour, Samsilah Roslan, Zeinab Ghiami, Mumtaz Ali Memon

**Affiliations:** ^1^Faculty of Health Sciences, University of Applied Sciences, Giessen, Germany; ^2^Faculty of Educational Studies, Universiti Putra Malaysia, Serdang, Malaysia; ^3^Faculty of Psychology and Sports Science, University of Münster, Münster, Germany; ^4^NUST Business School, National University of Sciences and Technology (NUST), Islamabad, Pakistan

**Keywords:** optimism, psychological well-being, self-efficacy, resilience, international students

## Abstract

The present study aims to examine whether self-efficacy mediates the relationship between optimism, dimensions of psychological well-being, and resilience among Iranian students. The participants in this study included 251 Iranian students from Universiti Putra Malaysia (UPM). Structural equation modeling using AMOS 20.0 was used to analyze the data. The results indicated that there were significant relationships between optimism, dimensions of psychological well-being, and resilience among Iranian students of UPM. The study findings presented that self-efficacy mediated the relationship between dimensions of psychological well-being (environmental mastery, autonomy, self-acceptance, positive relations with others, personal growth, and purpose in life) and resilience among Iranian students of UPM. Furthermore, self-efficacy was not observed to mediate the influence of optimism on resilience among Iranian students of UPM. The study’s findings help to understand the interrelationship between self-efficacy, various dimensions of psychological well-being, and resilience. Consequently, counselors, psychologists, and instructors can develop and plan valuable strategies to enhance students’ psychological factors.

## Introduction

International students face changes in every aspect of life, including geographical location, weather conditions, food, language, cultural habits, and behaviors (Mori, 2000; Titrek et al., 2016). Psychological problems (anxiety, stress, depression) among international students increase because of these changes (Takeuchi et al., 2007; Mesidor and sly, 2016). According to a study conducted by Talebloo and Baki (2013) among Universiti Putra Malaysia students from Iran, Yemen, and Saudi Arabia, faced various challenges during their studies. Furthermore, the Iranian Students’ Association in UPM (ISAM) has stated that Iranian students at Universiti Putra Malaysia face numerous problems and challenges. The main concerns of the students stem from financial issues, communication problems, and academic difficulties. In this regard, international students need to experience a period of adjustment to various educational and social situations. Therefore, students must understand the importance of adjustment and its related factors (Misra et al., 2003). In this respect, Mesidor and Sly (2016) indicated that the level of self-efficacy among international students was significantly related to students’ psychological adjustment.

Moreover, self-efficacy may play a role in how students feel about themselves, and whether they effectively accomplish their goals in life (Bartimote-Aufflick et al., 2016). Confidence in taking control over an individual’s behavior, social environment, and motivation can reflect self-efficacy (Bandura, 1977). Self-efficacy is a universal psychological need that controls an individual’s cognition, emotions, and decisions related to psychological well-being (Komarraju and Nadler, 2013). Consequently, self-efficacy is critical in stress management, influencing the stressor assessment, and the option and implementation of methods to deal with them (Villada et al., 2017). As a result, people who have high self-efficacy see potentially challenging situations as opportunities rather than risks (Liu and Li, 2018). Compared to people who have poor self-efficacy, they are more likely to use highly adaptive coping mechanisms (Chang and Edwards, 2015; Zhao et al., 2015). Hence, self-efficacy is an essential personal resource for university students to prevent stressors and promote adaptive adjustment to this formative stage (Morton et al., 2014; Denovan and Macaskill, 2017).

As Lightsey (2006) and Speight (2009) have reported, students’ self-efficacy is a significant predictor of their resilience. Moreover, resilience characteristics are essential to effectively cope with change, and the best predictor of adjustment to the new environment among international students (Wang, 2017). In this regard, Tusaie and Dyer (2004) expressed that resilient students could cope with the difficulties inherent in moving to a university in a foreign country, and succeed academically. Moreover, numerous researches suggest that resilience can be reinforce because it is not a “hard-wired” personality trait; instead, it is the consequence of the advancement of protective factors (Zimmerman and Arunkumar, 1994). Many, empirical studies reveal that resilience varies across individuals, and may highly depend on many other psychological factors, such as optimism and psychological well-being (Poyrazli et al., 2004; Rosenthal et al., 2007; Yusoff and Chelliah, 2010; Sagone and De Caroli, 2014).

Based on the review of previous studies on the mediating role of self-efficacy in the relationship between resilience and its predicting factors (Au et al., 2009; Souri and Hasanirad, 2011; Hosseinchari and Ghasem, 2012; Cody, 2013; Manganelli et al., 2015; Yu et al., 2015; Woldgabreal et al., 2016), no empirical study has examined the mediating role of self-efficacy in the relationship between optimism, psychological well-being, and resilience among international students in Malaysia. Although some models of self-efficacy and resiliency have been proposed, none of these models combined all these factors in a single study, and it is not known how this model fits the data in Asian countries. Therefore, the gaps that this study discusses are related to the lack of a quantitative explanation of mediating role of self-efficacy and its related factors among international students in a public university in Malaysia.

The specific objectives of this study are as follows:

1. To investigate the relationship between optimism, dimensions of psychological well-being and resilience among Iranian students in UPM; and
2. To investigate the mediating effect of self-efficacy in the relationship between optimism, dimensions of psychological well-being and resilience among Iranian students in UPM.

The research framework of this study provides access to a range of topics in resilience and self-efficacy. For the theoretical background of this research, resilience model of Kumper in conjunction with the social cognitive theory (SCT) was used. The resilience model of Kumpfer incorporates the predictive factors and process of resilience, leading to successful life adaptation in people who show resiliency. The Kumpfer model tries to clarify the variables known to multiply the resilience into a dynamic frame that describes the connections between an individual with high resilience and the individual’s high-risk context (Kumpfer, 2002).

To generate a classified framework to better understand personal factors related to resiliency, the internal individual factors are categorized into five main areas that may overlap: cognitive, emotional, social/behavioral, spiritual, and physical domains. The spiritual cluster of resiliency mainly involves cognitive competencies or belief structures. The variables are as follows: purpose in life, belief in uniqueness or oneself, independence, optimism, determination, and perseverance (Richardson et al., 1990). According to Kumpfer (2002), optimism is a cognitive factor, and highly resilient people apply optimistic characteristics to construct positive outcomes for themselves and others (Campbell et al., 1976; Murphy and Moriarty, 1976; Parker et al., 1990; Luthar and Zigler, 1991; Werner and Smith, 1992). A self-efficacy cycle comprises several successes developed by creating small steps with an excellent chance for success. Increasingly resilient youths are partial toward an “optimistic bias” and “latch on to any excuse for hope and faith in recovery” (Murphy, 1987). Other cognitive traits or processes that affect stimulate resilient people to positive success are believers in themselves and their specialness or uniqueness (Gordon and Song, 1994). Gordon and Song (1994) stated that achieving success despite all difficulties was related to autonomous self-directedness in resilient people. The authors described such individuals as autonomous/maverick selves who could resist social pressures and prefer to participate in goal-directed activities. The existential meaning and having a goal in life help resilient people pass through difficulties and become survivors because they have faith in achieving their goals (Richardson et al., 1990).

To succeed in their selected direction or mission, resilient persons are determined and perseverant in their cognitive style (Werner, 1985; Bandura, 1989). Life skills, practicality, talents, and competencies are necessary coping skills that help them reach their aims (Garmezy and Masten, 1986). Although determination and long-term planning skills are significant factors that help resilient people get their direction or mission, generating alternative or new plans and showing flexibility in planning are also essential aspects of resilient people (Bandura, 1989). Although behavioral and social competencies are similar to cognitive competencies because they build on the latter, these social and behavioral abilities vary due to the need for a behavioral action instead of only thinking. The social and behavioral ability to cope with diverse environments is referred to as “street smart” (Flach, 1988; Wolin and Wolin, 2010) or “environmental mastery” (Ryff, 1989), which is known to be related to resilience. Resilient people have warm, satisfying, trusting relationships with others (Ryff, 1989), and they have a sense of responsibility for other people (Werner, 1985). They show empathy for the needs of others, and also care for others (Werner, 1985). These individuals are active and responsive in relations with other people, and extract more positive reactions from them (Demos, 1989).

The resilient individual’s cognitive characteristics contain cognitive abilities that assist him/her in reaching his/her goals or dreams. Resilient young people can restore their resilient self-efficacy after disruption or failure in homeostasis (Bandura, 1977). Self-efficacy here is defined as the perceived ability to do a precise behavioral task (Bandura, 1977), perception of the effects of selecting challenges or duties, and emotional responses to the danger of failure (Lawrance and McLeroy, 1986). A youth who avoids testing challenges because of low self-esteem or self-efficacy will have a challenging time to improve his/her resilience (Schunk and Carbonari, 1984). Based on Bandura’s theory, undertaking challenges or defeating stressors is required to improve of self-efficacy. Shy or overprotected youths may not learn to face challenges due to the restrictive opportunities to increase their competencies and self-efficacy. Bandura also stated that individuals who experience only easy successes expect fast outcomes, and failure can quickly weaken their sense of efficacy (Bandura, 1989).

Social cognitive theory (SCT) includes a psychological model of behavior resulted from Albert Bandura’s effort (Bandura, 1986). Bandura (1986) improved the thinking concerning human functions to agree with the dominant role played by vicarious, cognitive, self-reflective, and self-regulatory processes in human change and adaptation. Based on this theoretical view, human functioning observes as an active interaction of environmental, behavioral, and personal effects. Among all the thoughts that stand at the very essence of social cognitive theory, and influence human functioning are self-efficacy beliefs: the judgment of individuals on their competencies to form and carry out actions to achieve a task. If individuals consider that their activities can yield the consequences they wish, they require little motivation to perform or deal with hardships. According to Bandura’s SCT, human functioning depends on an active interaction of environmental, behavioral, and personal effects. Self-efficacy beliefs affect the individuals’ choices and the sequences of acts that they follow. Furthermore, self-efficacy beliefs assist in regulating the extent of efforts individuals will exert to complete a task, the duration with which individuals face difficulties, and how they will confront the adverse circumstances. The highest sense of efficacy arises from the more significant effort, mastery, perseverance, motivation, and resilience (Bandura, 1989).

Individuals who have a greater sense of their competencies will approach difficult tasks as sophisticated challenges rather than threats to escape. They develop a better inherent concern and a profound obsession in their actions, tend to set themselves challenging aims, preserve a resilient commitment to these challenges, and increase and bear their efforts in the face of failure. Furthermore, they can recover their sense of efficacy more quickly after disappointments or obstacles, and attribute failure to inadequate action or a lack of information and abilities that they can acquire.

**Figure 1:**
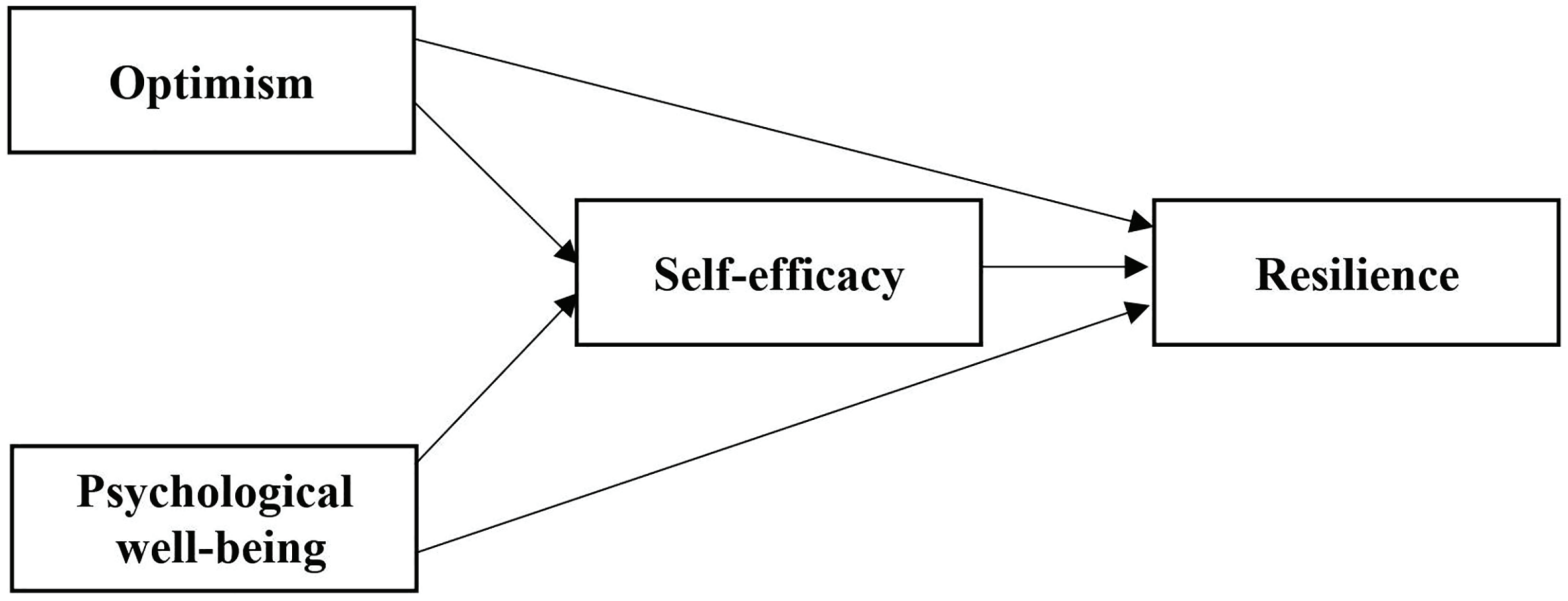
Conceptual framework.

To achieve the objectives of the study, a conceptual framework is designed (Figure 1).

## Literature Review

### Optimism, Psychological Well-Being, and Resilience

Carver et al. (2010) stated that optimism tended to imagine positive consequences in the future. Furthermore, when optimists encountered challenging but potentially surmountable obstacles, they tried to address the problems and focus on their goals, coping with problem-solving and careful planning (Carver et al., 2010). Optimistic individuals are more likely to show resilience when facing challenging situations even though they might show slow progress (Snyder et al., 2002). Miranda and Cruz (2020) indicated that optimism helped students cope with challenges in college. Furthermore, Dawson and Pooley (2013) revealed that students experience higher resilience levels with higher optimism levels. Optimistic individuals are more resilient when facing challenges than less optimistic individuals (Kleiman et al., 2017; Gómez Molinero et al., 2018; Pathak and Lata, 2018).

Based on the review of past literature, Ryff and Singer (2003) revealed that resilient persons could protect their physical and psychological well-being, and bounce back from tense events. As Acciari et al. (2019) stated, the levels of resilience and psychological well-being were interrelated. Ryff and Singer (2003) believed that maintaining mental and physical health, and the ability to recover faster from stressors characterize resilient individuals. Coward and Reed (1996) defined psychological well-being as an individual’s sensation of feeling healthy, resulting in a comprehensive consciousness of personal integrity that consisted of spiritual components of life. Previous studies indicated that psychological well-being (self-acceptance, environmental mastery, and personal growth) was significantly related to resilience (Keye and Pidgeon, 2013; Sagone and De Caroli, 2014).

Furthermore, a positive relationship between autonomy and resilience was revealed by Kaydkhorde et al. (2014). Based on Bernard (2014), environmental mastery, self-acceptance, and positive relation with others are significant resilience indicators. From this perspective the respondents perceived themselves as self-satisfied, and they were able to choose the contexts suitable for personal needs and see themselves as growing and expanding. Moreover, Picardi et al. (2012) reported a positive correlation between psychological well-being (except for the dimension of autonomy) and resilience.

*H1*: There is a significant relationship between optimism and resilience among Iranian students in UPM.

*H2*: There is a significant relationship between dimensions of psychological well-being and resilience among Iranian students in UPM.

### Self-Efficacy, Optimism, Psychological Well-Being, and Resilience

The association between optimism, psychological well-being, and resilience has been confirmed several studies (Dawson and Pooley, 2013; Bernard, 2014; Sagone and De Caroli, 2014), but little is known about the mediators of this association. Building upon the psychological perspective (Bandura, 1986; Kumpfer, 2002), the research focuses on the role of self-efficacy, as mediator.

Cervone (2004) proposed a new cognitive model of personality; in the model, self-efficacy represents knowledge about one’s abilities, which results in the appraisal of the future as positive (optimism), subsequently leading to good mental health. Karademas (2006) pointed out that high self-efficacy enhanced optimism and, subsequently, leads to better mental health; thus, optimism has been found to play a mediating role in the relationship between self-efficacy and depression (Karademas, 2006; Pu et al., 2016). Furthermore, the previous rationale and available literature showing that optimism contributes to self-efficacy (Karademas, 2006; Lee et al., 2006; Pu et al., 2016), and self-efficacy contributes to resilience (Speight, 2009; Keye and Pidgeon, 2013).

High self-efficacy is related to better well-being, and low self-efficacy is related to lower well-being levels (Caprara, 2002; Bandura et al., 2003). Significantly, positive relationships between psychological well-being and self-efficacy have also been reported (Bahadori Khosroshahi and Hashemi Nosrat Abad, 2012). Similarly, a significant positive relationship between academic self-efficacy and psychological well-being has also been observed (Asghari et al., 2014; Priesack and Alcock, 2015). Koller and Hicks (2016) found that mental health professionals in Australia would show higher psychological capital levels (self-efficacy, optimism, hope, and resilience). Moreover, they reported that this group showed higher psychological well-being levels (autonomy, environmental mastery, personal growth, relationship to others, purpose in life, self-acceptance).

Additionally, according to Choi et al. (2016), mastery experience is a prominent predictor of school principals’ self-efficacy in dealing with bullying among students in secondary schools. This result is in line with Bandura’s (1977) findings, which show that mastery experience is the essential determinant of self-efficacy. One of the inseparable components of the autonomy of students is their self-efficacy beliefs. Bandura (1982) declared that learners’ self-efficacy determined their performance since it affected their thoughts and motivation. Mojoudi and Tabatabaei (2014) revealed a relatively high correlation between upper intermediate students’ self-efficacy and autonomy, suggesting that boosting upper intermediate student’s self-efficacy will result in a considerable upturn in their autonomy.

Additionally, Tilfarlioglu and Ciftci (2011) found a positive correlation between self-efficacy and autonomy among students. Sanadgol (2015) investigated the relationship between self-acceptance and self-efficacy among high school students in Zahedan. The results supported the importance of self-acceptance as a predictor of self-efficacy. The sense of purpose in life has a significant meaning for well-being and self-efficacy. DeWitz et al. (2009) found a significantly positive correlation between self-efficacy and purpose in life. Çelik (2015) confirmed that the student academic support and academic self-efficacy are significantly correlated with personal growth initiative.

Hosseinchari and Ghasem (2012) demonstrated that intrinsic–extrinsic motivation could predict psychological resilience mediated by self-efficacy and stress appraisal. Similarly, a study of undergraduate students of Southeastern University in the United States discovered the mediating role of self-efficacy in a relationship between negative life changes and resilience (Cody, 2013). According to Hudson (2007), self-efficacy and parental engagements are two significant predictors of college students’ academic success and resilience. Hamill (2003) investigated self-efficacy, response to stress, perceptions of control, persistence, and coping mechanisms among maladaptive students and resilient students. The results indicated that self-efficacy and the capacity to articulate coping were two essential characteristics that distinguished the resilient group of the maladaptive youths, as predicted.

*H3*: There is a relationship between optimism and resilience among Iranian students in UPM when self-efficacy is mediated.

*H4*: There is a relationship between psychological well-being and resilience among Iranian students in UPM when self-efficacy is mediated.

## Materials and Methods

### Population, Sample Size, and Sampling Procedure

The population of this study is 841 Iranian students of UPM. The research employed GPower (version 3.1), with a significance level of 0.05, power of 0.80, and medium effect size (r = 0.30) to calculate the appropriate sample size. However, the study included a greater number of subjects to ensure the study’s reliability to counter the possibility of the nonresponse error. Indeed, for data collection procedures like surveys and other voluntary participation methods, the response rates are typically less than 100%. Therefore, oversampling was suggested by increasing the sample size for uncooperative subjects (Salkind and Rainwater, 2006). Additionally, Krejcie and Morgan (1970) indicated that to be more confident with the selected sample, acquiring a bigger sample than the numbers shown in their table of sample size is recommended.

Therefore, 400 samples out of 841 samples were selected from a sample frame of all Iranian students in UPM using a simple random sampling technique. Later, the researcher provided an online questionnaire form and emailed it to the targeted respondents. A total of 265 questionnaires were returned, out of which 251 questionnaires were filled appropriately for analysis in this study. Table 1 shows the distribution of the frequency and percentage by gender and marital status of the 251 respondents participated in this study. According to the results, 134 males (53.4%) and 117 females (46.6%) participated in this study. More than half of the respondents, 61.4% (*N* = 154), were single, and 38.6% (*N* = 97) were married.

**Table 1:** Profile of sample.

Variables (*n* = 291)	Frequency	Percentage (%)
Male	134	53.4
Female	117	46.6
Single	154	61.4
Married	97	38.6
Total	251	100

### Measures

To measure the variables of the study, four questionnaires were used. To measure resilience, the Connor-Davidson Resilience (CD-RISC) scale (Connor and Davidson, 2003), attempts to identify subjects’ capability in effectively dealing with stress and adversity was employed. The life orientation test – revised (LOT-R) was adopted from Scheier and Carver (1985) to assess optimism. LOT-R generalizes the outcome expectations and assumptions as stressed by Scheier and Carver’s optimism theory (Snyder et al., 2003). The General Self-Efficacy scale (Schwarzer and Jerusalem, 1995) was used to measure self-efficacy by predicting coping with daily stresses and adaptation after experiencing all kinds of stressful life events. To assess students’ psychological well-being, the Ryff Psychological Well-being scale was employed. The Ryff Psychological Well-Being scales was organized by Abbott et al. (2010) as a multidimensional concept to be a straightforward and relatively short survey that measures the psychological component of well-being.

### Data Characteristics

Preliminary analysis is an initial process at the beginning of a study to ensure the dataset is normally distributed. In this study, the preliminary analysis involved univariate normality and collinearity analysis. Kline (2015) suggested that the univariate normality of an item was achieved if the item’s skewness and kurtosis values range between −1.96 and +1.96. The results indicated that all the variables’ dimensions achieved the univariate normality (skewness and kurtosis values ranged from −1.193 to 1.177). Next, the collinearity diagnostic analysis was performed. According to Norusis (2012), the possibility of multicolinearity is low if the value of Tolerance is more than 0.1 while the value of VIF is less than 10.00. The value of Tolerance (range between 0.28 and 0.61) and variance inflation factor (range between 1.64 and 3.52) the requirement as recommended by Norusis (2012), indicating that the collinearity was not a severe issue in the present model.

### Confirmatory Factor Analysis

Confirmatory factor analysis (CFA) is a procedure to assess the measurement model by examining the relationship between observed variables and latent constructs (Brown, 2015). The CFA includes assessing factor loading, fit indices, and construct validity (e.g., convergent validity and discriminant validity). In general, factor loadings of 0.70 and above are considered satisfactory (Hair, 2010) and a factor loading of 0.6 is also suggested as acceptable (Chin et al., 1997). According to Table 2, after eliminating items with low factor loadings – less than 0.60 – the factor loadings for the remaining items ranged from 0.66 to 0.90.

**Table 2:** Results of measurement model assessment.

Construct	Item	Loadings	CR	AVE
Psychological well-being	PL01	0.761	0.892	0.734
	PL03	0.844		
	PL10	0.759		
	EN05	0.867		
	EN08	0.825		
	EN09	0.711		
	PG11	0.774		
	PG12	0.800		
	PG14	0.822		
	PR06	0.753		
	PR13	0.761		
	PR16	0.801		
	AU15	0.754		
	AU17	0.822		
	AU18	0.885		
	SA02	0.912		
	SA04	0.796		
	SA07	0.633		
Optimism	OP01	0.704	0.868	0.524
	OP02	0.794		
	OP03	0.710		
	OP05	0.646		
	OP07	0.707		
Resilience	R01	0.778	0.892	0.542
	R05	0.729		
	R09	0.692		
	R11	0.651		
	R12	0.767		
	R14	0.764		
	R15	0.762		
	R16	0.728		
	R17	0.865		
	R18	0.813		
	R19	0.765		
	R24	0.907		
	R25	0.820		

### Validity and Reliability

Convergent validity (CV) refers to the degree to which multiple items to measure the same concept agree (Hair et al., 2006). CV can be assessed through the average variance extracted (AVE) value. An AVE value of 0.5 confirms the CV of the construct. As shown in Table 2, the results indicated that the AVE for all variables exceeded the cut-off value of 0.05 – psychological well-being (0.734), optimism (0.524), and resilience (0.542) – thus, indicating that the constructs explained most of the variance (Hair, 2010). Furthermore, construct reliability was assessed to confirm the measures’ internal consistency through composite reliability (CR). A CR value of 0.7 and above proved that this study’s measures possessed satisfactory internal consistency reliability. The results of composite reliability showed that all constructs had achieved the standard criterion of psychological well-being (0.892), optimism (0.868), and resilience (0.892), indicating high consistency reliability of the instrument.

Besides that, Fornell and Larcker (1981) approach was used to assess the constructs’ discriminant validity. Discriminant validity ensures that a construct is genuinely distinct from other constructs in the model. The square root of AVE was compared with the correlations of latent variables. As a rule of thumb, AVE’s square must be greater than the correlation value of any other construct in the model (Fornell and Larcker, 1981). As shown in Table 3, the square root of AVE all constructs (diagonal values) was higher than the correlations values in the rows and column, suggesting that the measurement model had enough discriminant validity.

**Table 3:** Results of measurement model assessment.

	1	2	3	4	5	6	7	8	9
1. Resilience	**0.680**								
2. Self-efficacy	0.592	**0.738**							
3. Self-acceptance	0.005	0.054	**0.742**						
4. Purpose in life	0.353	0.442	0.157	**0.775**					
5. Environmental	−0.060	0.083	0.179	0.152	**0.793**				
6. Positive relation	0.417	0.438	0.065	0.313	0.127	**0.762**			
7. Personal growth	0.551	0.567	0.052	0.413	0.154	0.517	**0.767**		
8. Autonomy	0.207	0.177	0.097	0.199	0.316	0.213	0.267	**0.807**	
9. Optimism	0.583	0.531	−0.103	0.245	0.048	0.433	0.536	0.188	**0.784**

The bold values are the relationship values.

### Data Analysis

A two-stage structural equation modeling approach was employed to analyze the research model (Hair, 2010). The Analysis of Moment Structures (IBM AMOS) version 22 was used to test the hypothesized model.

## Results

**Figure 2:**
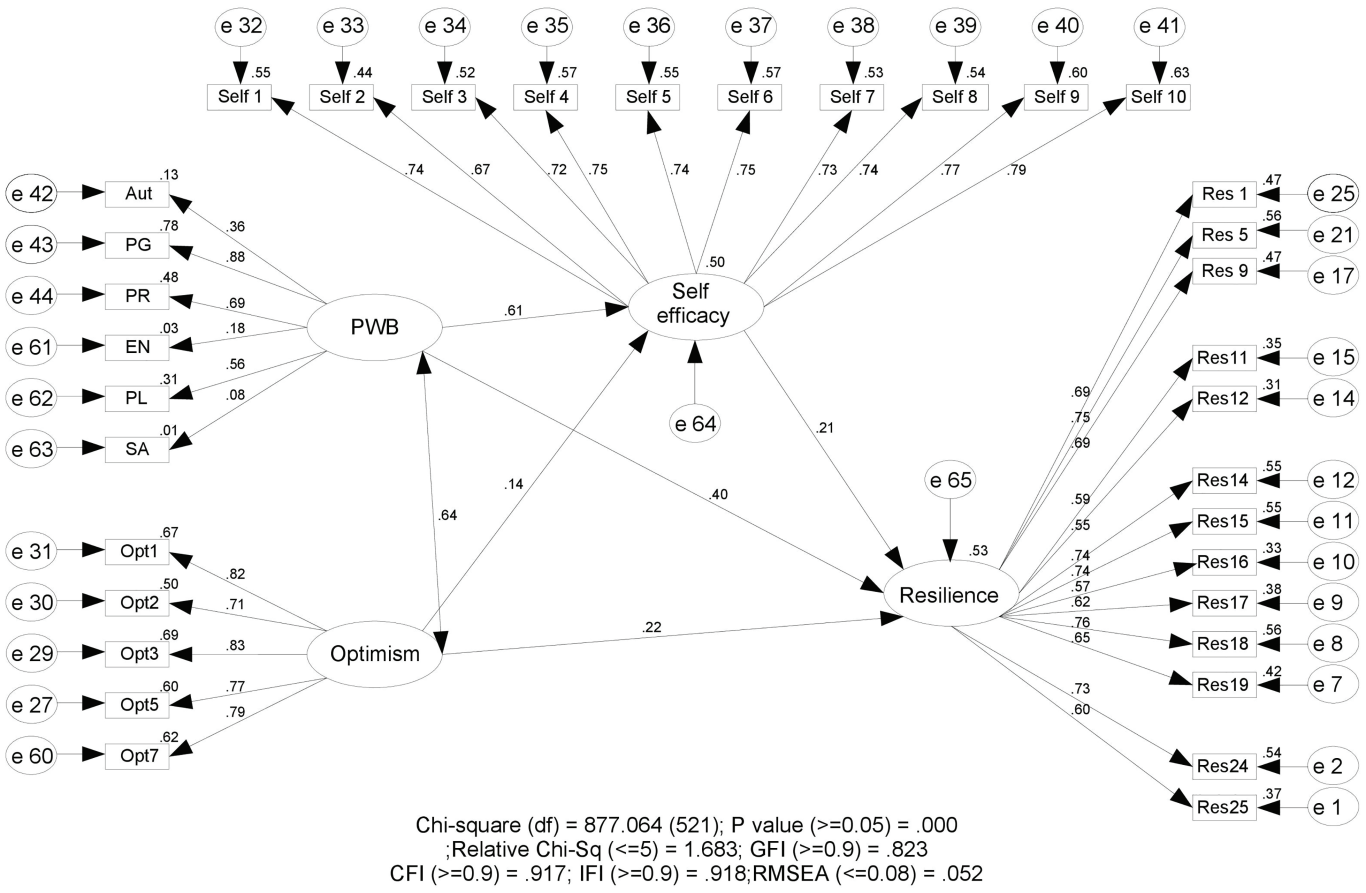
The full mediation structural equation model with standardized path coefficients.

The mediating effects of self-efficacy were assessed through path analysis. The full mediation structural model is displayed in Figure 2. The results of the full mediation structural model fit indicated that the model fit the data well, with Chi-square = 877.064, *p* = 0.000, Relative Chi-Sq = 1.683; GFI = 0.823, CFI = 0.917, IFI = 0.918, RMSEA = 0.052. The Goodness-of-fit indices of the structural model indicated that the GFI, CFI, and IFI approached or exceeded the cut-off value of 0.90 (Hair, 2010). Moreover, the RMSEA was 0.052, falling within the recommended range between 0.03 and 0.08 (Hair, 2010).

The regression weight of the total effect showed a significant impact of psychological well-being on resilience, and the insignificant impact of optimism on resilience (Table 4). In the next step, the bootstrapping procedure was conducted to test the mediation effects. Therefore, Bias-Corrected Confidence Interval (CI) was performed with a 95% confidence level and 5000 bootstraps. The outcomes of the bootstrap approximation were obtained by constructing two-sided bias-corrected confidence intervals. Table 5 indicates that the direct model (linking all dimensions of psychological well-being to resilience) showed a statistically significant effect (*β* = 0.549, *p* < 0.000). Moreover, the full mediation model (linking all dimensions of psychological well-being to resilience through self-efficacy) had a significant effect (*β* = 0.421, *p* < 0.000). The indirect impact of environmental mastery, autonomy, self-acceptance, positive relations with others, personal growth, and purpose in life on resilience was also significant. Furthermore, the direct model (linking optimism to resilience) was not statistically significant (*β* = 0.238, *p* < 0.000).

**Table 4:** Regression weight for the total effect.

Hypothesized relationship	B	β	S.E.	CR	*p*-value
PWB -> Resilience	1.036	0.549	0.254	4.074	0.000
Optimism -> Resilience	0.188	0.238	0.060	3.125	0.003

**Table 5:** Results of bootstrap analysis.

Hypothesis path	B	P	95% BC bootstrap CI	
			LB	UP
Direct model				
PWB -> Resilience	0.549	0.000		
Optimism -> Resilience	0.238	0.003		
Full mediation model				
PWB -> Resilience	0.421	0.000		
Std. indirect effects (SIE)	0.000	0.008	0.044	0.262
Optimism -> resilience	0.204	0.007		
Std. indirect effects (SIE)	0.030	0.150	−0.010	0.107

According to Table 5, zero is not within the range of lower and upper bounds. Therefore, based on the decision criteria, self-efficacy mediated the influence of environmental mastery, autonomy, self-acceptance, positive relations with others, personal growth, and purpose in life on resilience. Besides that, based on the decision criteria, self-efficacy did not mediate the influence of optimism on resilience.

## Discussion

This study examined the relationship between optimism, components of psychological well-being, and resilience among Iranian students in Universiti Putra Malaysia. Furthermore, the study assessed the mediating effect of self-efficacy on the relationship between optimism, components of psychological well-being, and resilience among Iranian students in Universiti Putra Malaysia.

The results showed a significant relationship between optimism, components of psychological well-being, and resilience, supporting hypotheses 1 and 2. The findings are in line with some other studies that observe the relationship between optimism, components of physiological well-being, and resilience (Agarwal and Malhotra, 2019; Bano and Pervaiz, 2020; Maheshwari and Jutta, 2020). It seems there is an interactive relationship between optimism, psychological well-being, and resilience. As the findings illustrate, students having a positive attitude are more likely to control their surroundings, and greater faith in their ability to respond to and resolve obstacles. In other words, positive attitudes about life and future improve resilience or the willingness to deal with hardship and difficult circumstances.

Furthermore, the findings revealed that in terms of shaping students’ perspectives on life issues, and their ability to handle and cope with problems, components of psychological well-being play an important role. This means that Iranian students seem to be self-satisfied; they can make relevant associations for their needs, and they see themselves as growing and expanding. Besides that, the students may have purposes and ambitions in their lives, such as being imaginative and efficient or achieving emotional integration later in life, both of which influence how they cope with challenging circumstances. The dimensions of psychological well-being affect the students’ perspectives on life issues, and their capability to manage and cope with challenges in their life (Smith and Yang, 2017; Chow et al., 2018).

The study’s finding well-supported H4, showing self-efficacy’s mediating role in the relationship between dimensions of psychological well-being (environmental mastery, autonomy, self-acceptance, positive relations with others, personal growth, and purpose in life) and resilience. The findings are consistent with the previous studies, confirming the relationship between psychological well-being, self-efficacy, and resilience, as well as the mediating role of self-efficacy (DeWitz et al., 2009; Tilfarlioglu and Ciftci, 2011; Mojoudi and Tabatabaei, 2014; Çelik, 2015; Sanadgol, 2015; Choi et al., 2016; Koller and Hicks, 2016; Ngui and Lay, 2017; Develos-Sacdalan and Bozkus, 2018; Wang et al., 2018; Roohi et al., 2019; Vongsirimas et al., 2020).

The findings can explain that the components of psychological well-being are related to self-efficacy, which would enhance resilience. It seems that the students’ belief in their ability has a significant effect on the students’ capability to maintain psychological and physical health and the ability to adjust to difficulties in the host country. Understanding this result is by considering the concept of self-efficacy as an adaptive mechanism, which refers to students’ belief in their capability to fulfill tasks to achieve their goals. In this respect, through educational programs, students’ self-efficacy can improve, which has a significant role in increasing psychological well-being and resilience.

### Implication

Theoretically, consistent with Kumpfer’s (2002) and Bandura’s (1986) findings, this study supported the notion of a relationship between optimism, dimensions of psychological well-being, self-efficacy, and resilience. This study has conducted a comprehensive review based on the theory and constructs that are less explored in this topic’s research theory. The result have led to the development of a new model of variables that contribute to resilience with the mediating role of self-efficacy among Iranian students. The model provides complete knowledge of the topic while simplifying the understanding of the situation for both researchers and practitioners. This research is a systematic attempt to contribute to a deeper understanding of these theories, although there is still room to improve and add more knowledge to this stream.

Practically, the research on self-efficacy and resilience has revealed that there has been a tendency for the psychology discipline to neglect crucial human adaptation and development. It instead focuses on potential; this approach should change. Hopefully, the findings of this study provide an overview of the challenges faced by Iranian students, and support them accordingly. The notion of self-efficacy and resilience has significant implications in educational psychology in five main domains: assessment, intervention, consultation, research, and training. Since self-efficacy and resilience, and their related factors can be learned and taught as a skill, the authorities in charge of international students in universities and organizations are responsible for regulating the policies to enhance students’ ability to deal with the challenges. Therefore, related informative workshops and seminars should improve students’ knowledge and understanding concerning self-efficacy, resilience, and relevant factors. In this respect, there are various projects and strategies available to develop, nurture and teach skills. For instance, self-efficacy training for international students (SETIS) is based on social cognitive theory, which is adequate in describing this phenomenon and recommending strategies to improve students’ self-efficacy. The implementation of SETIS meets a standard procedure for performing successful training, including a need assessment and a post-training assessment. Moreover, a training program called the Road to Resilience developed by American Psychological Association trains students to build resilience or “strengthen the mental muscle that everyone has,” using “bounce back” strategies. These may include: have a friend and be a friend, set new goals and plan to reach them, take charge of your behavior, look on the bright side, and believe in yourself.

Understanding the strategies to develop self-efficacy and resiliency as skills would help international students to reduce the time required for the adjustment process. For example, Bandura (1977) indicated, self-efficacy beliefs were created when students perceive knowledge from four main sources. (1) Mastery Experience: the way students perceive and analyze obtained outcomes, as well as how these interpretations were used to update and establish self-beliefs of competence. (2) Vicarious Experiences of observing others: seeing or hearing similar individuals achieve success encourages students to feel that if others can do it, so can they. (3) Modeling Experiences: those are the people students look up to, respect, and aspire to be like. Their deeds, values, and accomplishments teach and persuade students to do the same. (4) Emotional and Physical Experiences: self-efficacy is strongly affected by student’s mental and physical states. Positive experiences and happiness make students feel good about themselves. In contrast, negative experiences and tension make students feel insecure. Students should be aware of the strategies to enhance their self-efficacy and apply them in their lifestyle and attitude. This study demonstrates the self-efficacy and resilience pattern to help alleviate some of the students’ challenges and social–emotional adjustment. The awareness can encourage students to successfully deal with unfamiliar situations throughout not only their education, but also their life.

Besides that, it is necessary for counselors, educators, and professionals to consider the evidence obtained from the current study to design self-efficacy and resilience enhancement programs for international students. Hence, these findings can help to increase the availability of the program through counseling services. Appropriate counseling opportunities should be given to international students facing difficulties adjusting to new situations. Moreover, the findings can be used as a resource material for researchers, scientists, and university authorities. This study also opens the doors for further research on self-efficacy and resilience, and its related factors to discover new findings that could be generalized to all international students.

### Limitation and Future Suggestions

In the future, applying more qualitative and longitudinal researches could help to investigate how instructional strategies can be implemented to promote international students’ self-efficacy and resilience. The findings may be more generalizable by recruiting a more comprehensive sample size from different nationalities in future studies.

## Data Availability Statement

The data that support the findings of this study are available from the first author upon reasonable request. Requests to access the datasets should be directed to f.sabouripour@gmx.de.

## Ethics Statement

The studies involving human participants were reviewed and approved by Graduated Student Office, Universiti Putra Malaysia. The patients/participants provided their written informed consent to participate in this study.

## Author Contributions

We declare that all authors have made substantial contributions. FS was in charge of the conceptualization, investigation, methodology, writing original the draft, editing, and formatting the paper. SR was supervising the study. ZG was involved in the methodology and formal analysis. MAM was involved in the formal analysis. All authors approved the submitted version.

### Conflict of Interest

The authors declare that the research was conducted in the absence of any commercial or financial relationships that could be construed as a potential conflict of interest.
